# Journalistic

**Published:** 1874-02

**Authors:** 


### JOURNALISTIC.

"*The Pennsylvania Journal of Dental Science.*"

A monthly record of the proceedings of the dental societies, and of dental science in general. Edited and published by Sam'l Welchens, D. D. S., Lancaster, Penn."

The first number of this journal was issued Jan. 1, 1874, and makes an excellent start so far as matter and appearance is concerned. It is well gotten up, and especially so if, as the editor says, it sprung from a mere contemplation to a completion within six weeks. If this is only a foretaste, and that under disadvantages, what shall we have when the editor gets fairly settled in his harness.

We welcome the Penna. Journal into the ranks of our professional periodicals. There is room. There is much that is valuable that hitherto our journals have not been able to publish ; an increase of journals will not only gather up much that would not otherwise be published, but will be an additional stimulus to the profession to write.

